# A multisite validation of a two hours antibiotic susceptibility flow cytometry assay directly from positive blood cultures

**DOI:** 10.1186/s12866-024-03341-1

**Published:** 2024-05-28

**Authors:** C. Pina-Vaz, A. Silva-Dias, I. Martins-Oliveira, R. Gomes, B. Perez-Viso, S. Cruz, A. G. Rodrigues, A. Sarmento, R. Cantón

**Affiliations:** 1 FASTinov SA, UPTEC Science and Technology Campus, Porto, Portugal; 2 Division of Microbiology, Department of Pathology, Faculty of Medicine, University of Porto, Porto, Portugal; 3 CINTESIS/RISE-Center for Health Technology and Services Research, Faculty of Medicine, University of Porto, Porto, Portugal; 4 Servicio de Microbiología, Hospital Universitario Ramón y Cajal and Instituto Ramón y Cajal de Investigación Sanitaria (IRYCIS), Madrid, Spain; 5 Department of Infectious Diseases, Centro Hospitalar de São João, Porto, Portugal; 6 CIBER de Enfermedades Infecciosas (CIBERINFEC), Instituto de Salud Carlos III, Madrid, Spain

**Keywords:** Rapid antimicrobial susceptibility test, Flow cytometry, Antimicrobial resistance, Blood cultures, Bloodstream infections

## Abstract

**Background:**

Rapid antimicrobial susceptibility testing (AST) is urgently needed to provide safer treatment to counteract antimicrobial resistance. This is critical in septic patients, because resistance increases empiric therapy uncertainty and the risk of a poor outcome. We validate a novel 2h flow cytometry AST assay directly from positive blood cultures (PBC) by using a room temperature stable FASTgramneg and FASTgrampos kits (FASTinov® Porto, Portugal) in three sites: FASTinov (site-1), Hospital Ramon y Cajal, Madrid, Spain (site-2) and Centro Hospitalar S. João, Porto, Portugal (site-3). A total of 670 PBC were included: 333 spiked (site-1) and 337 clinical PBC (151 site-2 and 186 site-3): 367 gram-negative and 303 gram-positive. Manufacturer instructions were followed for sample preparation, panel inoculation, incubation (1h/37ºC) and flow cytometry analysis using CytoFlex (Site-1 and -2) or DxFlex (site-3) both instruments from Beckman-Coulter, USA.

**Results:**

A proprietary software (bioFAST) was used to immediately generate a susceptibility report in less than 2 h. In parallel, samples were processed according to reference AST methods (disk diffusion and/or microdilution) and interpreted with EUCAST and CLSI criteria. Additionally, ten samples were spiked in all sites for inter-laboratory reproducibility. Sensitivity and specificity were >95% for all antimicrobials. Reproducibility was 96.8%/95.0% for FASTgramneg and 95.1%/95.1% for FASTgrampos regarding EUCAST/CLSI criteria, respectively.

**Conclusion:**

FASTinov® kits consistently provide ultra-rapid AST in 2h with high accuracy and reproducibility on both Gram-negative and Gram-positive bacteria. This technology creates a new paradigm in bacterial infection management and holds the potential to significantly impact septic patient outcomes and antimicrobial stewardship.

**Supplementary Information:**

The online version contains supplementary material available at 10.1186/s12866-024-03341-1.

## Background

Sepsis has been recognized worldwide as the most common cause of death with an estimated 11 million each year in 48.9 million episodes [1]. The current standard diagnostic method of bloodstream infections is blood culture (BC) to determine the etiology of the bacteremia, ideally collected before antimicrobials are administered to the patients. According to international guidelines, empiric therapy should be initiated within an hour from recognition of sepsis [2] as delay is associated with increased mortality among adults and neonates [3, 4]. Furthermore, the initial empirical therapy can be inappropriate in up to 50% of cases [5, 6] with impact on patient morbidity, mortality, length of stay, and cost of care [7]. Due to the increase of antimicrobial resistance (AMR) [1] and the frightening estimations for next decades [8] empiric therapy is not anymore safe as it could fail in 20% of the cases [9].

Standard antimicrobial susceptibility testing (AST) might require 24–48 h given it is growth-dependent, which delays the use of targeted therapy and timely escalation/de-escalation [10]. Advances are needed to decrease the time-to-result (TTR) for the diagnosis of bloodstream infections as rapid identification and susceptibility report can aid in the potentially lifesaving administration of targeted antimicrobial therapy. Ultra-rapid identification methods directly from BC are now available such as matrix-assisted laser desorption ionization–time of flight mass spectrometry (MALDI-TOF) [11], Filmarray [12] or ePlex [13]. Some of them have additionally the ability to identify genes associated with specific resistance mechanisms, but resistance might be too complex to drive prescription based only on molecular assays. Moreover, the absence of genes associated with resistance does not mean susceptibility as gene expression might be influenced by several conditions that may increase or even silence resistance genes. Therefore, when generated under a Time-To-Response similar to that of molecular/proteomic methods, rapid phenotypic susceptibility analysis may be a better option. It provides the benefit of exposing the bacteria to antimicrobials and then studying its behavior individually. These phenotypic assays could provide safer and more precise and effective information for patient treatment.

Recent approaches have been introduced with the capacity to detect early bacterial growth such as the Accelerate Pheno [14], the QuickMIC [15], ASTar system [16], Quantamatrix [17] or Specific Reveal [18] methods. All of them are growth-dependent like standard AST, taking in general 5–8 h to provide results, depending on the microorganism and/or phenotype; it is faster to determine resistance than susceptibility on those kind of growth-base assays. Currently, only Accelerate and Quantamatrix include susceptibility for both gram-negative and gram-positive bacteria approved for clinical use. EUCAST recently presented a rapid method, the RAST, using disk diffusion from PBCs, providing results after 4, 6 and 8 h incubation of the plates depending on the bacterial isolate [19].

FASTinov is presenting a growth-independent, ultra-rapid (2 h), phenotypic disruptive technology based on flow cytometry. It is used to perform a multiparametric analysis of bacteria that are incubated for a short 1 h with antimicrobial drugs and a fluorescent probe. Using specific drugs at breakpoint concentrations, categorical interpretations (susceptible [S], susceptible increased exposure or intermediate [I], susceptible dose-dependent [SDD] or resistant [R]) based on either EUCAST or CLSI criteria are performed by a proprietary software. Whenever needed, it may also provide MIC values using serial concentrations of a certain drug, already demonstrated for vancomycin [20] and colistin [21]. The FASTinov report can be released in up to 2 h after a PBC flags positive. This is actually the most rapid phenotypic susceptibility, and gives the physician timely guidance to move from empirical therapy to targeted therapy often even before the second dose of antibiotic. The objective of this study is to perform a validation of the FASTinov® dehydrated panels FASTgramneg and FASTgrampos (CE-IVD) for antimicrobial susceptibility assay (AST) directly from PBC performed in 3 sites: an internal validation on FASTinov laboratory (Porto, Portugal) with spiked blood cultures and a clinical validation with PBC obtained from patients admitted in two hospitals (Ramón y Cajal University Hospital in Madrid, Spain, and Centro Hospitalar S. João in Porto, Portugal).

## Methods

### Study design and sample collection

All sites included in this study used BACTEC blood bottles (Becton Dickinson, US) BC. Site-1, FASTinov, used spiked BC with well characterized strains belonging to FASTinov bacterial collection as well as quality control strains (described in additional file 1) between January 2021 and May 2023; they were incubated until they flagged positive. Site-2, Ramón y Cajal University Hospital, a university public health center with around 1,000 beds, included sequential patients’ PBC, one from each patient, between February and June, 2021. Site-3, Centro Hospitalar S. João (CHSJ), an university hospital with around 1,000 beds, included sequential patients’ PBC, one from each patient between December 2021 and July 2022.

Microorganisms were identified from PBC by MALDI-TOF (Bruker Daltonics, Germany). A large diversity of species was recovered. All tested isolates were sub-cultured on blood agar plates to assess purity and bacterial identification was assessed with MALDI-TOF. Antimicrobial susceptibility was determined using standardized methods; the strains were frozen at -80ºC with the approved study codification. Polymicrobial BC were excluded.

### Ethical considerations

The study was approved by the corresponding ethical committees of the Ramón y Cajal University Hospital (reference no. 161/17) and Centro Hospitalar S. João (reference no. 284/21).

### FASTinov assay

PBC already identified by Bruker MALDI Biotyper CA System (Bruker Daltonics, DE) and processed according to the instructions for use (IFUs) of the FASTinov® kit, which include four steps: (1) Sample preparation: Extract 1 ml of the BC bottle, mix with a haemolytic agent (tergitol 10%), centrifuge for 1 min at 13,000 rpm; this procedure will lyse red blood cells. Resuspend the pellet with 1 ml of sterile saline solution, add 500 µl of this suspension to 500 µl of Histopaque® (a density gradient medium) and centrifuge again at the same speed and duration; this achieves removal of remaining blood components, preserving the bacteria. Resuspend pellet in 500 µl of sterile saline solution, prepare a 0.5 MacFarland suspension and dilute 1 ml in 7 ml of Muller-Hinton cation adjusted broth medium (26177). When performed by a trained technician, the whole sequence takes max. 5–10 min. (2) Inoculation and incubation of the panels: FASTinov panels are dried, room temperature 96 well microplates with a panel that includes the main antimicrobials used in clinic settings for sepsis together with a fluorescent probe previously optimized [20]. The FASTinov assay is a breakpoint test, with some concentrations of each drug present on the panel, except in the case of vancomycin for which enough concentrations are included to allow MIC determination in the case of *Staphylococcus aureus*. Additionally, the kits detect the presence of extended spectrum beta-lactamases (ESBL) on Enterobacterales group-I (*E. coli*, *Klebsiella* spp, *P. mirabilis*, *Salmonella spp*., *Shigella spp*) and screen for the possible presence of ESBL for Enterobacterales of group-II (*Enterobacter spp, Citrobacter freudii, Morganella morganii, Providencia stuarti, Serratia* spp, *Hafnia alvei*), pAmpC or carbapenemases, according the EUCAST protocol for detection of mechanisms of resistance [22]. The panels are inoculated with 100 µl of the bacterial suspension using a multichannel pipette and subsequently incubated for 60 min at 37ºC with shaking; (3) Analysis by flow cytometry: To evaluate cell lesions triggered by antibiotic exposure, a flow cytometric fully automated analysis was performed using CytoFlex model B3-R0-V3 (Beckman Coulter, USA) at site-1 and site-2 and DxFlex (Beckman Coulter, USA) at site-3, both equipped with one blue laser (488 nm; output, 50 mW; beam spot size, 5 by 80 μm). The instruments have three fluorescence channels: 525/40 BP, 585/42 BP, and 690/50 BP and are also equipped with a plate reader for the automatic analysis of each panel. The flow cytometers were used on slow mode; and (4) Software analysis: A dedicated software was used for data analysis and results were compared with the ones obtained with standardized (disk diffusion) and/or standard methods (ISO broth microdilution). Expert rules were included in the software [23].

### Time to results (TTR) and instrument time (IT)

TTR is defined as the sum of the durations of the four steps described above: (1) sample prep, (2) incubation, (3) flow cytometry instrument time (IT) and (4) software report. There is a fixed duration time for sample prep and incubation; duration time of flow cytometry instrument depends on the number of wells analyzed (drugs and concentrations) which vary with the bacterial species and the reference protocol (EUCAST or CLSI) (see Table 6).

### Reproducibility

At least 10 determinations for each antibiotic were performed in triplicate at FASTinov with strains mostly with MIC on scale allowing the calculation of inter-laboratorial reproducibility.

### Reference antimicrobial susceptibility testing method and interpretation

PBC were sub-cultured in blood agar and growth colonies were identified by MALDI-TOF and submitted to AST assay according to the reference disk diffusion and/or broth microdilution techniques. The results were analyzed by a different operator, using the EUCAST [23] and CLSI [24] breakpoints tables.

### Data analysis and discrepancy resolution

The S, I, S-DD and R interpretive category results obtained with the FASTinov assays were compared to those of the reference methods. Sensitivity and specificity of both kits according the standard definition of ISO 20776-2 respectively for EUCAST and CLSI protocols was determined [23–25] Additionally, bias, categorical agreement (CA)- agreement of interpretative results (SIR) between the FASTinov results and the reference methodand essential agreement (EA)- agreement within plus or minus, one two-fold dilution of the FASTinov assay with the reference method MIC determination were calculated, and errors quantified and classified as minor (mE)- minor discrepancies (the reference category result is R or S and the new device result is I; or the reference result is I and the new device result is R or S)/total organisms testedx100, major (ME)- major discrepancies (the reference category result is S and the new device result is R)/total susceptibility organisms by reference method, and very major (VME)- very major discrepancies (the reference category result is R and the new device result is S)/total resistant organisms by reference method. The proportion of agreement (PA) and the sensitivity and specificity for detecting ESBL in Enterobacterales group-I was determined; screening for the presence of pAmpC, carbapenemases, and ESBL in *Enterobacterales* group-II was also calculated. Susceptibility is evaluated comparing several cellular parameters of treated cells with breakpoint concentrations compared to non-treated cells (control); If they present morphology changes and/or increase in the intensity of fluorescence (meaning cell damage) they are considered susceptible. If treated cells look like the control, means resistance. MIC determination by FASTinov technology was calculated as the lowest concentration of the drug that showed susceptibility.

Any discrepant result, despite the kind of error associated, was repeated and if persists broth microdilution performed.

## Results

Distribution by species of the total 670 PBC studied (367 Gram-negative and 303 Gram-positive) is represented on Table 1 (Gram-negative and Gram-positive). Results per site, can be consulted in additional files [2–7].

**Table 1:** Distribution per site of tested isolates in FASTgramneg kit and FASTgrampos kit

***Gram-negative bacteria***				
	**Site 1**	**Site 2**	**Site 3**	**Total**
**Enterobacterales**	**100**	**76**	**76**	**252**
* Escherichia coli*	17	49	38	104
* Klebsiella pneumoniae*	56	13	25	94
* Klebsiella aerogenes*	4	1	2	7
* Klebsiella oxytoca*	-	2	1	3
* Kluyvera variicola*	-	-	1	1
* Enterobacter cloacae*	11	3	-	14
* Enterobacter kobei*	-	1	-	1
* Citrobacter koseri*	-	2	-	2
* Citrobacter freundii*	-	1	-	1
* Proteus mirabilis*	5	4	4	13
* Serratia marcescens*	3	-	2	5
* Serratia nematodiphila*	-	-	1	1
* Morganella morganii*	-	-	2	2
* Salmonella enteritidis*	3	-	-	3
* Providencia rettgeri*	1	-	-	1
**Non-fermenters**	**102**	**7**	**6**	**115**
* Pseudomonas aeruginosa*	72	5	5	82
* Acinetobacter baumanii*	30	-	-	30
* Acinetobacter calcoaceticus*	-	1	-	1
* Acinetobacterpitti*	-	1	-	1
* Acinetobactervariabilis*	-	-	1	1
**Total**	**202**	**83**	**82**	**367**
***Gram-positive bacteria***				
	**Site 1**	**Site 2**	**Site 3**	**Total**
***Staphylococcus *spp**	**66**	**46**	**92**	**204**
* Staphylococcus aureus*	35	12	16	63
* Staphylococcus epidermidis*	23	24	41	88
* Staphylococcus capitis*	-	1	12	13
* Staphylococcus hominis*	8	7	17	32
* Staphylococcus haemolyticus*	-	1	3	4
* Staphylococcus simulans*	-	-	1	1
* Staphylococcus lugdunensis*	-	1	1	2
* Staphylococcus warneri*	-	-	1	1
***Enterococcus *spp**	**65**	**22**	**12**	**99**
* Enterococcus faecalis*	44	10	7	61
* Enterococcus faecium*	18	9	5	32
* Enterococcus gallinarum*	2	2	-	4
* Enterococcus casseiliflavus*	1	-	-	1
* Enterococcus raffinosus*	-	1	-	1
**Total**	**131**	**68**	**104**	**303**

Site-1: FASTinov laboratory, Porto; Site-2: Hospital Ramón y Cajal, Madrid; Site-3: Centro Hospitalar S. João, Porto.

### FASTinov kits performance

Based on analysis of the global results with the EUCAST/CLSI guidelines, the sensitivity and specificity of the test was superior to 96% as shown on Table 2. The FAST*gramneg* kit achieved overall CA ≥ 97% with errors < 1.5% (Table 3). Regarding amoxicillin/clavulanic acid (EUCAST), ceftazidime/avibactam and amikacin (both on EUCAST/CLSI) the CA was 100%.

**Table 2:** Sensitivity and specificity of the FASTinov® kits according EUCAST and CLSI protocols when compared to reference method

**FAST*gramneg***	**EUCAST/CLSI**	**Site 1**	**Site 2**	**Site 3**	**Total**
	**Sensitivity (%)**	99.0/99.0	97.7/96.6	98.5/100	98.7/98.8
	**Specificity (%)**	97.9/96.6	99.5/97.5	99.5/98.8	98.9/97.5
**FAST*grampos***	**Sensitivity (%)**	100/100	98.5/99.0	100/100	99.8/99.8
	**Specificity (%)**	96.9/96.8	97.5/97.6	97.5/97.5	97.3/97.2

Site-1: FASTinov, Porto; Site-2: Hospital Ramón y Cajal, Madrid; Site-3: Centro Hospitalar S. João, Porto

**Table 3:** FASTgramneg results obtained with total strains of 3 sites compared with reference methods (RM) (for results per site see additional file)

**FASTgramneg**																
		**EUCAST**							**CLSI**							
		**RM**							**RM**							
**Antimicrobial agent**	n	I	R	CA(%)	mE	ME	VME	n	S	I	SDD	R	CA(%)	mE	ME	VME
**Ampicillin**	250	-	184	99.6	-	-	1/184	250	66	1	-	183	99.2	2/250	-	-
**Amoxacillin-clavulanic acid**	250	-	144	100	-	-	-	250	110	14	-	126	97.2	7/250	-	-
**Cefotaxime**	250	1	92	98.4	1/250	3/157	-	250	157	1	-	92	98.0	3/250	2/157	-
**Ceftazidime**	331	35	135	98.5	1/331	2/161	1/135	332	198	4	-	130	97.3	5/332	1/198	3/130
**Cefepime**	332	52	112	98.5	2/332	1/168	1/112	333	216	8	5	104	96.0	10/333	-	3/104
**Piperacillin-tazobactam**	333	51	105	97.3	-	5/177	4/105	364	237	15	-	112	94.8	13/364	5/237	1/112
**Ceftolozane-tazobactam**	331	-	61	97.3		4/270	5/61	331	270	5	-	56	96.0	6/331	3/270	4/56
**Ceftazidime-avibactam**	334	-	11	100	-	-	-	334	323	-	-	11	100	-	-	-
**Meropenem**	251	2	20	98.4	3/251	-	1/20	251	219	9	-	23	98.4	3/251	-	1/23
**Ciprofloxacin**	366	55	150	98.9	2/366	2/161	-	366	215	6	-	145	98.9	3/366	1/215	-
**Gentamicin**	282	-	77	99.3	-	1/205	1/77	364	273	6	-	85	99.5	2/364	-	-
**Amikacin**	362	-	31	100	-	-	-	362	334	-	-	28	100	-	-	-
Overall	3672	196	1122	98.9	0.2%	0.80%	1.2%	3787	2618	69	5	1095	97.9	1.4%	0.5%	1.1%

Fifty-three strains of Enterobacterales group-I were ESBL positive when using reference methods and FASTinov assay had sensitivity and specificity of 96.2% and 100% respectively, with a PA of 99.0%. Regarding screening for ESBL on Enterobacterales of group-II, 13 were positive being the sensitivity and specificity 100%, with a PA of 100% too. For plasmid AmpC screening (Enterobacterales group-I), 38 strains were positive on reference methods, and sensitivity, specificity and PA were 100%. Overall, 67 isolates were positive in the carbapenemase screening (meropenem MIC > 0.25 mg/L) and FASTinov test showed 97.1% sensitivity (2 false negative results were found in isolates displaying meropenem MICs of 0.5 mg/L) and 96.7% specificity with a PA of 96.8%.

The Gram-positive kits showed CA > 97% (Table 4). All tested drugs showed a CA > 94%. The ME were 2.4–2.5% specially regarding *Enterococcus* and vancomycin and gentamicin.

**Table 4:** FAST*grampos* results obtained with total strains of 3 sites compared with reference methods (RM) (for results per site see supplementary data)

**FASTgrampos**		EUCAST										CLSI							
		RM										RM							
Antimicrobial agent	*n*	**S**	I	*R*		EA(%)	CA(%)	mE	ME	VME	*n*	S	I	*R*	EA(%)	CA(%)	mE	ME	VME
Penicillin*	75	19	-	56		-	94.7	-	3/19	1/56	303	94	-	209	-	98.3	-	4/94	1/209
**Ampicillin**	99	59	2	38		-	98.0	2/99	-	-	99	61	-	38	-	98.9	-	1/61	-
Cefoxitin**	100	48	-	52		-	96.0	-	4/48	-	100	48	-	52	-	96.0	-	4/48	-
Oxacillin**	88	14	-	74		-	95.5	-	4/14	-	88	14	-	74	-	95.5	-	4/14	-
**Vancomycin**	265	254	-	11		100	98.9	-	3/254	-	265	254	3	8	100	98.9	-	3/254	-
**Linezolid**	303	298	-	5		-	98.3	2/303	3/298	-	303	298	-	5	-	98.0	3/303	3/298	-
**Gentamicin**	201	131	-	70		-	99.0	-	2/131	-	201	147	-	54	-	98.5	1/201	2/147	-
**Gentamicin high level**	66	51	-	15		-	95.5	-	3/51	-	66	51	-	15	-	96.9	-	2/51	-
**Overall**	1197	874	2	321			97.7	0.3%	2.5%	0.3%	1425	967	3	455		98.0	0.3%	2.4%	0.2%

Cefoxitin**- except *S. epidermidis*

Oxacillin***- only *S. epidermidis*

Penicillin*- only for *S. aureus* on EUCAST

**Fig. 1:**
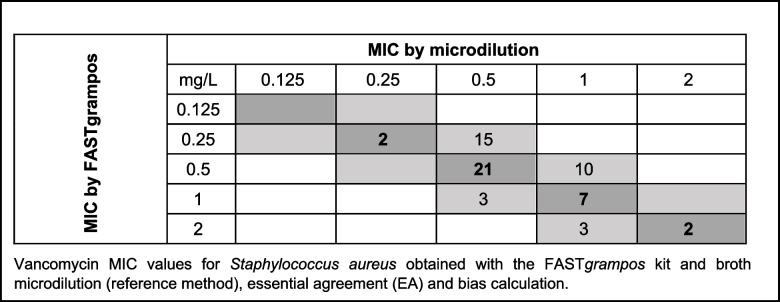
Distribution of vancomycin MIC values

 The EA for MIC determination of vancomycin on *S. aureus* was 100%, being negative bias of -30% (which is on the inferior limit accepted by ISO 20776-2:2021) [25]. Figure 1 represents the distribution of vancomycin MICs obtained in *S. aureus* tested isolates. Note that the strains that gave 1 dilution lower on the FASTgrampos assay comparing to microdilution, were the strains with MIC values of 0.5 ug/ml or 1 ug/ml, not on breakpoint concentrations, with no impact on treatment.

Regarding VME, detail is presented on Table 5. Higher number (5 strains) was observed with ceftalozane/tazobactam. Two strains (EB033 and EB069) presented more than one error and several were ESBL positive. Only 1 VME was observed with Gram-positive kit.

**Table 5:** Very Major Errors (VME) obtained with FASTgramneg and FASTgrampos kits according to EUCAST and CLSI

**Drug**	***n***	**Strain**	**Identification**	**MIC (mg/L)**	**Protocol**	**Resistance mechanism**	**Site**
Ampicillin	1	EB 106	*E. coli*	16	EUCAST*		2
	3	EB 033	*K. pneumoniae*	32	EUCAST/CLSI*	ESBL	2
		EB 052	*E. coli*	64	CLSI	ESBL	2
		K098	*K. pneumoniae*	32	CLSI*	KPC	1
Ceftazidime	4	EB 1168	*E. cloacae*	32	EUCAST/CLSI*		1
		EB 033	*k. pneumoniae*	32	CLSI*	ESBL	2
		EB 069	*K. aerogenes*	16	CLSI*	ESBL	2
Piperacillin/ tazobactam	4	EB 101	*k. pneumoniae*	64/4	EUCAST	ESBL	1
		EB 123	*E. cloacae*	64/4	EUCAST		1
		EB 718	*E. coli*	> 128/4	EUCAST/CLSI*	ESBL, KPC	1
		EB 069	*K. aerogenes*	64/4	EUCAST	ESBL	2
Ceftalozane/ tazobactam	5	EB 101	*K. pneumoniae*	> 32/4	EUCAST/CLSI	ESBL	1
		EB 677	*K. pneumoniae*	16/4	EUCAST/CLSI*	ESBL	1
		EB 679	*K. pneumoniae*	> 32/4	EUCAST/CLSI	ESBL	1
		EB 032	*K. pneumoniae*	4/4	EUCAST*	ESBL	3
		EB161	*E.coli*	> 64/4	EUCAST/CLSI	KPC	1
Meropenem	1	EB 021	*K. pneumoniae*	16	EUCAST*/CLSI	ESBL	2
Gentamicin	1	EB 286	*E. coli*	8	EUCAST*		3
Penicillin	1	ST 099	*S. aureus*	0.25	EUCAST*/CLSI*		2

Site of origin (Site-1: FASTinov, Porto; Site-2: Hospital Ramón y Cajal, Madrid; site-3: Centro Hospitalar S. João, Porto)

*One dilution distance from breakpoint concentration

### Reproducibility

Reproducibility regarding FASTgramneg was 96.8%/95% and for FASTgrampos was 95.1%/95.1% when using EUCAST/CLSI criteria.

### Instrument time and time-to-results

The time taken by the instrument to analyze each microorganism was recorded on Table 6. The minimum time was 9 min for *Acinetobacter* spp on EUCAST protocol and the highest, 47 min with *Enterobacterales* under CLSI. Instrument time depends on the number of drugs and concentrations tested for each microorganism and the selected protocol. Since incubation always takes 60 min and the sample prep takes no more than 10 min, TTR had a minimum of 79 min, and maximum of 117 min, and all tests were performed in less than 2 h.

**Table 6:** Time of instrument. Cytometer reading time for FASTinov panels

**Type of bacteria**	EUCAST	CLSI
*Enterobacterales*	39 min	47 min
*Pseudomonas* spp	15 min	27 min
*Acinetobacter* spp	9 min	23 min
*Staphylococcus* spp	27 min	29 min
*Enterococcus* spp	17 min	20 min

## Discussion

This study demonstrates that FASTinov 2 h AST assay, now using dried CE-marked panels, are ultra-rapid and accurate which is in agreement with the results published earlier using frozen panels [20]. The results were similar for both Gram-negative and Gram-positive bacteria including a considerable number of species (16 species of *Enterobacterales*, *Pseudomonas aeruginosa* and 3 species of *Acinetobacter*; 8 species of *Staphylococcus* and 5 of *Enterococcus*). The number of antibiotics tested was also wide, 12 for *Enterobacterales*, 9 for *Pseudomonas* spp., 4 for *Acinetobacter* spp. in case of FAST*gramneg* kit; 5 for *Staphylococcus* spp. and *Enterococcus* spp. regarding FAST*grampos*. This study included spiked BC in order to have a broader variety of phenotypes but also clinical samples with.

TTR inferior to 2 h in all cases regardless of the microorganism and/or phenotype. Same-shift workflows in labs, will allow communication to the clinician on time to drive same-shift therapy adjustments. Delays on appropriate treatment specially more than 6 h correlates with increase 30-days mortality [26]. This could prove the 2 h-AST from FASTinov as unique when compared to other rapid technologies that provide results in 5–8 h, which inevitably causes results to be communicated in latter shifts and driving therapy changes next day only.

ESBL producers were detected with great accuracy. Most of the errors were with beta-lactam antibiotics; Less than 1.5% of VME were found, especially on ESBL producers but most of them presenting MIC values close to breakpoint.

The ability to provide information regarding possible presence of other enzymatic resistance mechanisms recommended by EUCAST, namely ESBL on Enterobacterales group-II, pAmpC or carbapenemases, is also of value. In case of a positive screening result, confirmation should be performed using another test. Regarding pAmpC, we recently described a flow cytometry assay also taking less than 2 h [27]. The detection of the underlined mechanisms of resistance is relevant not only for patient treatment but also for public health and even for the development of new drugs and/or inhibitors.

FASTinov sample prep is fast but yet manual. Future developments may automate this step, but not at the expense of increasing the overall TTR. Automation must produce a clean suspension of viable bacteria needed by flow cytometry to achieve high accuracy.

The 2 h AST from FASTinov has proven also the ability to rapidly perform MIC determinations, but each drug needs to be analyzed individually over an array of concentrations, which increases reading time at a rate around 1.5 min per well. One limitation of the assay is that only one panel is analyzed each time. This is why the time of instrument is relevant. Increasing instrument time, currently 10–50 min, would reduce the number of samples that can be analyzed per day.

The new technology is designed to transform the management of bacteremia. It is intended to offer cost-effective solutions for top-tier healthcare facilities managing sepsis patients at risk of severe bacterial infections, aligning in general with comparable technologies.

The clinical benefit of this ultra-rapid AST is clear, especially for critically ill patients with bacterial infection. Further evidence collected from real-world use should show the clinical and economic benefits to sepsis patients, as well as rational and safe use of antimicrobial therapies.

## Conclusion

This disruptive technology has great potential to change the antimicrobial therapy, not only on sepsis patients, but also on future development areas such as urinary tract infections and others. To achieve its full benefit, pre-lab processes and lab communication to clinicians must be optimal. In summary, we conclude that FASTinov technology is ultra-rapid, accurate and reproducible, competing with molecular assays in terms of speed while providing the clearest therapy guidance from phenotypic susceptibility.

### Supplementary Information

Supplementary Material 1.

Supplementary Material 2.

Supplementary Material 3.

Supplementary Material 4.

Supplementary Material 5.

Supplementary Material 6.

Supplementary Material 7.

## Data Availability

More details of data are summarized on additional files.

## Abbreviations

AMR: Antimicrobial resistance
AST: Antimicrobial susceptibility test
BC: Blood culture
CA: Categorical agreement
CLSI: Clinical and Laboratory Standards Institute
EA: Essential agreement
ESBL: Extended spectrum beta-lactamases
EUCAST: European committee on antimicrobial susceptibility testing
I: Intermediate
ISO: International organization for standardization
ME: Major error
mE: minor Error
MIC: Minimal inhibitory concentration
PA: Proportion of agreement
PBC: Positive blood culture
R: Resistant
S-DD: Susceptible dose-dependent
S: Susceptible
VME: Very major error

## References

[1] Collaborators AR (2022). Global burden of bacterial antimicrobial resistance in 2019: a systematic analysis. Lancet.

[2] Rhodes A, Evans LE, Alhazzani W, Levy MM, Antonelli M, Ferrer R (2017). Surviving sepsis campaign: international guidelines for management of sepsis and septic shock: 2016. Intensive Care Med.

[3] Kumar A, Roberts D, Wood KE, Light B, Parrillo JE, Sharma S (2006). Duration of hypotension before initiation of effective antimicrobial therapy is the critical determinant of survival in human septic shock. Crit Care Med.

[4] Apisarnthanarak A, Holzmann-Pazgal G, Hamvas A, Olsen MA, Fraser VJ (2004). Antimicrobial use and the influence of inadequate empiric antimicrobial therapy on the outcomes of nosocomial bloodstream infections in a neonatal intensive care unit. Infect Control Hosp Epidemiol.

[5] Herzke CA, Chen LF, Anderson DJ, Choi Y, Sexton DJ, Kaye KS (2009). Empirical antimicrobial therapy for bloodstream infection due to methicillin-resistant Staphylococcus aureus: no better than a coin toss. Infect Control Hosp Epidemiol.

[6] Ibrahim EH, Sherman G, Ward S, Fraser VJ, Kollef MH (2000). The influence of inadequate antimicrobial treatment of bloodstream infections on patient outcomes in the ICU setting. Chest.

[7] Kaye KS, Marchaim D, Chen TY, Baures T, Anderson DJ, Choi Y (2014). Effect of nosocomial bloodstream infections on mortality, length of stay, and hospital costs in older adults. J Am Geriatr Soc.

[8] de Kraker ME, Stewardson AJ, Harbarth S (2016). Will 10 million people die a year due to antimicrobial resistance by 2050?. PLoS Med.

[9] Kumar A, Ellis P, Arabi Y, Roberts D, Light B, Parrillo JE (2009). Initiation of inappropriate antimicrobial therapy results in a fivefold reduction of survival in human septic shock. Chest.

[10] Buehler SS, Madison B, Snyder SR, Derzon JH, Cornish NE, Saubolle MA (2016). Effectiveness of practices to increase timeliness of providing targeted therapy for inpatients with bloodstream infections: a laboratory medicine best practices systematic review and meta-analysis. Clin Microbiol Rev.

[11] Bhatti MM, Boonlayangoor S, Beavis KG, Tesic V (2014). Rapid identification of positive blood cultures by matrix-assisted laser desorption ionization-time of flight mass spectrometry using prewarmed agar plates. J Clin Microbiol.

[12] Bhatti MM, Boonlayangoor S, Beavis KG, Tesic V (2014). Evaluation of FilmArray and Verigene systems for rapid identification of positive blood cultures. J Clin Microbiol.

[13] Huang TD, Melnik E, Bogaerts P, Evrard S, Glupczynski Y (2019). Evaluation of the ePlex blood culture identification panels for detection of pathogens in bloodstream infections. J Clin Microbiol.

[14] Charnot-Katsikas A, Tesic V, Love N, Hill B, Bethel C, Boonlayangoor S (2018). Use of the accelerate pheno system for identification and antimicrobial susceptibility testing of pathogens in positive blood cultures and impact on time to results and workflow. J Clin Microbiol.

[15] Malmberg C, Torpner J, Fernberg J, Öhrn H, Ångström J, Johansson C (2022). Evaluation of the speed, accuracy and precision of the QuickMIC rapid antibiotic susceptibility testing assay with gram-negative bacteria in a clinical setting. Front Cell Infect Microbiol.

[16] Göransson J, Sundqvist M, Ghaderi E, Lisby JG, Molin Y, Eriksson E et al. Performance of a system for Rapid phenotypic Antimicrobial susceptibility testing of Gram-negative Bacteria directly from positive blood culture bottles. J Clin Microbiol. 2023;61(3):e0152522. 10.1128/jcm.01525-22 PMC10035315 36852983

[17] Rosselin M, Prod'hom G, Greub G, Croxatto A (2022). Performance evaluation of the Quantamatrix QMAC-dRAST system for rapid antibiotic susceptibility testing directly from blood cultures. Microorganisms.

[18] Tibbetts R, George S, Burwell R, Rajeev L, Rhodes PA, Singh P (2022). Performance of the reveal rapid antibiotic susceptibility testing system on gram-negative blood cultures at a large urban hospital. J Clin Microbiol.

[19] Jonasson E, Matuschek E, Kahlmeter G (2020). The EUCAST rapid disc diffusion method for antimicrobial susceptibility testing directly from positive blood culture bottles. J Antimicrob Chemother.

[20] Silva-Dias A, Pérez-Viso B, Martins-Oliveira I, Gomes R, Rodrigues AG, Cantón R (2021). Evaluation of FASTinov ultrarapid flow cytometry antimicrobial susceptibility testing directly from positive blood cultures. J Clin Microbiol.

[21] Fonseca ESD, Silva-Dias A, Gomes R, Martins-Oliveira I, Ramos MH, Rodrigues AG, et al. Evaluation of rapid colistin susceptibility directly from positive blood cultures using a flow cytometry assay. Int J Antimicrob Agents. 2019;54(6):820–3. 10.1016/j.ijantimicag.2019.08.016 31425793

[22] Diseases ESoCMaI. EUCAST guidelines for detection of resistance mechanisms and specific resistances of clinical and/or epidemiological importance 2017 V.2.0. Available from: https://www.eucast.org/fileadmin/src/media/PDFs/EUCAST_files/Resistance_mechanisms/EUCAST_detection_of_resistance_mechanisms_170711.pdf.

[23] Testing ECoAS. European committee on antimicrobial susceptibility testing breakpoint tables for interpretation of MICs and zone diameters version 9.0, valid from 2019-01-01 ed. 2023.

[24] Institute CaLS (2022). M100 performance standards for antimicrobial susceptibility testing. 32nd ed.

[25] Susceptibility testing of infectious agents and evaluation of performance of antimicrobial susceptibility test devices ISO. 2021;20776–2:2021.

[26] Van Heuverswyn J, Valik JK, van der Desirée S, Hedberg P, Giske C, Nauclér P (2023). Association between time to appropriate antimicrobial treatment and 30-day mortality in patients with bloodstream infections: a retrospective cohort study. Clin Infect Dis.

[27] Martins-Oliveira I, Pérez-Viso B, Silva-Dias A, Gomes R, Peixe L, Novais Â (2022). Rapid detection of plasmid AmpC beta-lactamases by a flow cytometry assay. Antibiotics (Basel).

