# Supplementary material for: A multisite validation of a two hours antibiotic susceptibility flow cytometry assay directly from positive blood cultures
Source: BMC Microbiol. 2024 May 28;24:187. doi: 10.1186/s12866-024-03341-1 (PMC11131321; doi:10.1186/s12866-024-03341-1)
Supplement: Supplementary file 1 — Supplementary Material 1. [file 12866_2024_3341_MOESM1_ESM.pdf]

Additional file 1. Reference strains used in the validation of FASTinov panels.

**Reference strains**	**Mechanism of resistance**
Enterobacter aerogenes ATCC 13048	Wild type
Enterobacter cloacae CCUG 59627	AmpC beta-lactamase hyperproducer
Escherichia coli NCTC 13846	Colistin resistant. MCR-1 positive
Escherichia coli ATCC 25922	Wild type
Escherichia coli ATCC 35218	Ampicillin resistant
Escherichia coli ATCC 8739	Wild type
Escherichia coli BAA 2452	Metallo-beta-lactamase (NDM-1) producer
Escherichia coli NCTC 13476	IMP-type metallo-beta-lactamase producer
Klebsiella pneumoniae ATCC 700603	SHV-18 beta-lactamase producer
Klebsiella pneumoniae BAA 1705	Carbapenemase (KPC) producer
Klebsiella pneumoniae BAA 1706	Non carbapenemase producer
Klebsiella pneumoniae NCTC 13443	NDM-1 producer
Pseudomonas aeruginosa ATCC 27853	Wild type
Providencia rettgeri BAA 2525	Carbapenemase (OXA-48) producer
Serratia marcescens ATCC 14756	Wild type
Staphylococcus aureus ATCC 29213	Methicillin susceptible
Staphylococcus epidermidis ATCC 35984	---
Staphylococcus aureus ATCC 43300	Methicillin resistant
Enterococcus faecalis ATCC 29212	---
Enterococcus faecalis ATCC 51299	*vanB*
Enterococcus faecium ATCC 700221	*vanA*
Enterococcus casseliflavus ATCC 700668	*vanC-2/3,*
Enterococcus gallinarum ATCC 49608	*vanC-1*
