# Supplementary material for: A multisite validation of a two hours antibiotic susceptibility flow cytometry assay directly from positive blood cultures
Source: BMC Microbiol. 2024 May 28;24:187. doi: 10.1186/s12866-024-03341-1 (PMC11131321; doi:10.1186/s12866-024-03341-1)
Supplement: Supplementary file 2 — Supplementary Material 2. [file 12866_2024_3341_MOESM2_ESM.pdf]

Additional file 2. FAST*gramneg* results obtained with total strains of site 1 compared with reference methods

**FASTgramneg: total spiked on blood cultures FASTinov, site 1**																	
		**EUCAST**							**CLSI**								
		**RM**								**RM**							
**Antimicrobial agent**	**n**	**S**	**I**	**R**	**CA(%)**	**mE**	**ME**	**VME**	**n**	**S**	**I**	**SDD**	**R**	**CA(%)**	**mE**	**ME**	**VME**
**Ampicillin**	100	14	-	86	100	-	-	-	100	81	-	-	86	100	-	-	-
**Amoxacillin-clavulanic acid**	100	19	-	81	100	-	-	-	100	81	5	-	75	97	3/100	-	-
**Cefotaxime**	100	28	1	71	97	1/100	2/28	-	100	28	1	-	71	97	1/100	2/28	-
**Ceftazidime**	172	28	30	114	97.7	-	2/28	1/114	172	60	3	-	109	98.8	-	1/60	1/109
**Cefepime**	172	35	46	91	98.3	1/172	1/35	-	172	78	5	2	87	96.5	5/172	-	1/87
**Piperacillin-tazobactam**	172	33	49	90	95.3	-	5/33	3/90	202	90	8	-	104	93.6	7/202	5/90	1/104
**Ceftolozane-tazobactam**	172	115	-	57	95.3	-	4/115	4/57	172	115	4	-	53	93.0	5/172	3/115	4/53
**Ceftazidime-avibactam**	172	161	-	11	100	-	-	-	172	161	-	-	11	100	-	-	-
**Meropenem**	100	81	2	17	97	3/100	-	-	100	71	9	-	20	97	3/100	-	-
**Ciprofloxacin**	202	41	48	113	99.5	1/202		-	202	89	3	-	110	99.5	1/202	-	-
**Gentamicin**	130	75	-	55	100	-	-	-	202	131	3	-	68	99.5	1/202	-	-
**Amikacin**	202	174	-	28	100	-	-	-	202	176	-	-	26	100	-	-	-
**Overall**	1794	804	176	814	98.4	0.3%	1.7%	0.98%	1896	1161	41	2	820	97.7	1,40%	0.95%	0.85%
