# Supplementary material for: A multisite validation of a two hours antibiotic susceptibility flow cytometry assay directly from positive blood cultures
Source: BMC Microbiol. 2024 May 28;24:187. doi: 10.1186/s12866-024-03341-1 (PMC11131321; doi:10.1186/s12866-024-03341-1)
Supplement: Supplementary file 3 — Supplementary Material 3. [file 12866_2024_3341_MOESM3_ESM.pdf]

Additional file 3. FAST*gramneg* results obtained with total strains of site 2 compared with reference methods

***FASTgramneg* Total of blood cultures Hospital Ramon Y Cajal, site 2**	**EUCAST**								**CLSI**									
	**RM**								**RM**									
**Antimicrobial agent**	**n**	**S**	**I**	**R**	**CA(%)**	**mE**	**ME**	**VME**	**n**	**S**	**I**	**SDD**	**R**	**CA(%)**	**mE**	**ME**	**VME**
**Ampicillin**	74	20	-	54	98.6	-	-	1/54	74	20	1	-	53	97.3	2/74	-	-
**Amoxacillin-clavulanic acid**	74	44	-	30	100	-	-	-	74	46	9	-	19	94.6	4/74	-	-
**Cefotaxime**	74	61	-	13	98.7	-	1/61	-	74	61	-	-	13	96.0	2/74	-	-
**Ceftazidime**	78	60	3	15	100	-	-	-	78	64	1	-	13	92.3	4/78	-	2/13
**Cefepime**	79	62	5	12	98.7	-	-	1/12	79	66	-	3	10	93.7	3/79	-	2/10
**Piperacillin-tazobactam**	80	69	2	9	98.8	-	-	1/9	80	71	7	-	2	96.3	3/80	-	-
**Ceftolozane-tazobactam**	78	75	-	3	100	-	-	-	78	75	-	-	3	100	-	-	-
**Ceftazidime-avibactam**	81	81	-	-	100	-	-	-	81	81	-	-	-	100	-	-	-
**Meropenem**	75	72	-	3	98.7	-		1/3	75	72	-	-	3	98.7	-		1/3
**Ciprofloxacin**	82	53	7	22	98.8	1/82	-	-	82	59	3	-	20	98.8	1/82	-	-
**Gentamicin**	75	65	-	10	97.3	-	2/65	-	80	70	1	-	9	100	-	-	-
**Amikacin**	78	77	-	1	100	-	-	-	78	77	-	-	1	100	-	-	-
**Overall**	928	739	17	172	99.1	0.1%	0.4%	2.3%	933	762	22	3	146	97.5	2.0%	-	3.4%
