# Supplementary material for: A multisite validation of a two hours antibiotic susceptibility flow cytometry assay directly from positive blood cultures
Source: BMC Microbiol. 2024 May 28;24:187. doi: 10.1186/s12866-024-03341-1 (PMC11131321; doi:10.1186/s12866-024-03341-1)
Supplement: Supplementary file 4 — Supplementary Material 4. [file 12866_2024_3341_MOESM4_ESM.pdf]

Additional file 4. FAST*gramneg* results obtained with total strains of site 3 compared with reference methods

FASTgramneg **Total of blood cultures Centro Hospitalar S. João, site 3**	**EUCAST**								**CLSI**							
	**RM**								**RM**							
**Antimicrobial agent**	**n**	**S**	**I**	**R**	**CA(%)**	**mE**	**ME**	**VME**	**n**	**S**	**I**	**R**	**CA(%)**	**mE**	**ME**	**VME**
**Ampicillin**	76	32	-	44	100	-	-	-	76	32	-	44	100	-	-	-
**Amoxacillin-clavulanic acid**	76	43	-	33	100	-		-	76	44	-	32	100	-		-
**Cefotaxime**	76	68	-	8	100	-	-	-	76	68	-	8	100	-	-	-
**Ceftazidime**	81	73	2	6	98.8	1/81	-	-	82	74	-	8	98.8	1/82	-	-
**Cefepime**	81	71	1	9	98.8	1/81	-	-	82	72	3	7	97.6	2/82	-	-
**Piperacillin-tazobactam**	81	75	-	6	100	-	-	-	82	76	-	6	96.3	3/82	-	-
**Ceftolozane-tazobactam**	81	80	-	1	98.7	-	-	1/1	81	80	1	-	98.8	1/81	-	-
**Ceftazidime-avibactam**	81	81	-	-	100	-	-	-	81	81	-	-	100	-	-	-
**Meropenem**	76	76	-	-	100	-	-	-	76	76	-	-	100	-	-	-
**Ciprofloxacin**	82	67	-	15	97.6	-	2/67	-	82	67	-	15	97.6	1/82	1/67	-
**Gentamicin**	77	65	-	12	98.7	-	-	1/12	82	72	2	8	98.8	1/82	-	-
**Amikacin**	82	80	-	2	100	-	-	-	82	81	-	1	100	-	-	-
**Overall**	950	811	3	136	99.4	0.2%	0.2%	1.5%	958	823	6	129	99.1	0.9%	0.1%	-
