# Supplementary material for: A multisite validation of a two hours antibiotic susceptibility flow cytometry assay directly from positive blood cultures
Source: BMC Microbiol. 2024 May 28;24:187. doi: 10.1186/s12866-024-03341-1 (PMC11131321; doi:10.1186/s12866-024-03341-1)
Supplement: Supplementary file 5 — Supplementary Material 5. [file 12866_2024_3341_MOESM5_ESM.pdf]

Additional file 5. FAST*grampos* results obtained with total strains of site 1 compared with reference methods

FASTgrampos total of blood cultures	EUCAST										CLSI									
FASTinov, site 1	RM										RM									
**Antimicrobial agent**	**n**	**S**	**I**	**R**	**EA(%)**	**CA(%)**	**mE**	**ME**	**VME**		**n**	**S**	**I**	**R**	**EA(%)**	**CA(%)**	**mE**	**ME**	**VME**	
**Penicillin***	35	7	-	28	-	94.3	-	2/7	-		131	49	-	82	-	97.7	-	3/49	-	
**Ampicillin**	65	42	-	23	-	98.5	1/65	-	-		65	42	-	23	-	100	-	-	-	
**Cefoxitin****	43	26	-	17	-	95.3	-	2/26	-		43	26	-	17	-	95.3	-	2/26	-	
**Oxacillin***- only S. epidermidis**	23	4	-	19	-	91.3	-	2/4	-		23	4	-	19	-	91.3	-	2/4	-	
**Imipenem**	65	-	39	26	-	100	-	-	-		NA	NA	NA	NA	-	-	-	-	-	
**Vancomycin**	110	100	-	10	100	98.2	-	2/100	-		110	100	3	7	100	98.2	-	2/100	-	
**Linezolid**	131	128	-	3	-	99.2	-	1/128	-		131	128	-	3	-	98.5	1/131	1/128	-	
**Gentamicin**	66	37	-	29	-	98.5	-	1/37	-		66	51	-	15	-	97.0	1/66	1/51	-	
**Gentamicin high level**	47	35	-	12	-	95.5	-	2/35	-		47	35	-	12	-	97.9	-	1/35	-	
**Overall**	585	379	39	167	100	97.8	0.1%	3.2%	0.0%		616	435	3	178	100	97.7	0.3%	2.8%	0.0%	

Penicillin*- only for *S. aureus* on EUCAST

Cefoxitin**- except *S. epidermidis*

Oxacillin***- only *S. epidermidis*
