# Supplementary material for: A multisite validation of a two hours antibiotic susceptibility flow cytometry assay directly from positive blood cultures
Source: BMC Microbiol. 2024 May 28;24:187. doi: 10.1186/s12866-024-03341-1 (PMC11131321; doi:10.1186/s12866-024-03341-1)
Supplement: Supplementary file 6 — Supplementary Material 6. [file 12866_2024_3341_MOESM6_ESM.pdf]

**Additional file 6**. FAST*grampos* results obtained with total strains of site 2 compared with reference methods

| FASTgrampos: total of patient's | EUCAST | | | | | | | | CLSI | | | | | | | |
|---|---|---|---|---|---|---|---|---|---|---|---|---|---|---|---|---|
| Hospital Ramon Y Cajal, site2 | RM | | | | | | | | RM | | | | | | | |
| Antimicrobial agent | n | S | I | R | CA(%) | mE | ME | VME | n | S | I | R | CA(%) | mE | ME | VME |
| Penicillin* | 12 | 2 | - | 10 | 91.7 | - | - | 1/10 | 68 | 15 | - | 53 | 98.5 | - | - | 1/53 |
| Ampicillin | 22 | 10 | 2 | 10 | 95.5 | 1/22 | - | - | 22 | 12 | - | 10 | 95.5 | - | 1/12 | - |
| Cefoxitin** | 22 | 13 | - | 9 | 95.5 | - | 1/13 | - | 22 | 13 | - | 9 | 95.5 | - | 1/13 | - |
| Oxacillin** | 24 | 5 | - | 19 | 100 | - | - | - | 24 | 5 | - | 19 | 100 | - | - | - |
| Imipenem | 13 | - | 12 | 1 | 100 | - | - | - | NA | NA | NA | NA | - | - | - | - |
| Vancomycin | 56 | 56 | - | - | 100 | - | - | - | 56 | 56 | - | - | 100 | - | - | - |
| Linezolid | 68 | 68 | - | - | 97,1 | - | 2/68 | - | 68 | 68 | - | - | 97.1 | - | 2/68 | - |
| Gentamicin | 43 | 26 | - | 17 | 97.7 | - | 1/26 | - | 43 | 28 | - | 15 | 97.7 | - | 1/28 | - |
| Gentamicin high level | 12 | 10 | - | 2 | 100 | - | - | - | 12 | 10 | - | 2 | 100 | - | - | - |
| **Overall** | 272 | 190 | 14 | 68 | 97.8 | 0.4% | 2.1% | 1.5% | 315 | 207 | - | 108 | 98.1 | 0.0% | 2.4% | 0.9% |

Penicillin*- only for *S. aureus* on EUCAST

Cefoxitin**- except *S. epidermidis*

Oxacillin***- only *S. epidermidis*
