# Supplementary material for: A multisite validation of a two hours antibiotic susceptibility flow cytometry assay directly from positive blood cultures
Source: BMC Microbiol. 2024 May 28;24:187. doi: 10.1186/s12866-024-03341-1 (PMC11131321; doi:10.1186/s12866-024-03341-1)
Supplement: Supplementary file 7 — Supplementary Material 7. [file 12866_2024_3341_MOESM7_ESM.pdf]

Additional file 7. FAST*grampos* results obtained with total strains of site 3 compared with reference methods

FASTgrampos total of blood cultures	EUCAST									CLSI								
**Centro Hospitalar S. João, site 3**	RM									RM								
**Antimicrobial agent**	**n**	**S**	**I**	**R**	**EA(%)**	**CA(%)**	**mE**	**ME**	**VME**	**n**	**S**	**I**	**R**	**EA(%)**	**CA(%)**	**mE**	**ME**	**VME**
**Penicillin***	28	10	-	18	-	96.4	-	1/10	-	104	30	-	74		99.0	-	1/30	-
**Ampicillin**	12	7	-	5	-	100	-	-	-	12	7	-	5		100	-	-	-
**Cefoxitin****	35	9	-	26	-	97.1	-	1/9	-	35	9	-	26		97.1	-	1/9	-
**Oxacillin****	41	5	-	36	-	95.1	-	2/5	-	41	5	-	36		95.1	-	2/5	-
**Vancomycin**	99	98	-	1	100	99.0	-	1/98	-	99	98	-	1	100	98.9	-	1/98	-
**Linezolid**	104	102	-	2	-	98.1	2/104	-	-	104	102	-	2		98.1	2/104	-	-
**Gentamicin**	92	68	-	24	-	100	-	-	-	92	68	-	24		100	-	-	-
**Gentamicin high level**	7	6	-	1	-	85.7	-	1/6	-	7	6	-	1		85.7	-	1/6	-
**Overall**	418	305	-	113	-	98.1	0.5%	1.9%	-	494	325	-	169		98.4	0.4%	1.8%	-

Penicillin*- only for *S. aureus* on EUCAST

Cefoxitin**- except *S. epidermidis*

Oxacillin***- only *S. epidermidis*
